# A New Approach in Part Design for Friction Stir Welding of 3D-Printed Parts with Different Infill Ratios and Colors

**DOI:** 10.3390/polym16131790

**Published:** 2024-06-25

**Authors:** Oğuz Koçar, Nergizhan Anaç, Erhan Baysal

**Affiliations:** 1 Department of Mechanical Engineering, Faculty of Engineering, Zonguldak Bülent Ecevit University, Zonguldak 67100, Türkiye; oguz.kocar@yahoo.com.tr; 2 Alaplı Vocational School, Zonguldak Bülent Ecevit University, Zonguldak 67850, Türkiye; erhanbaysal@beun.edu.tr

**Keywords:** 3D printing, infill ratio, friction stir welding, color measurement, heat distribution

## Abstract

Parts produced using a 3D printer are combined with friction stir welding (FSW). In the FSW processing of parts with a low infill ratio, welding errors occur due to a lack of material. In this study, plates were created using two different-colored PLA Plus filaments with different infill ratios in the weld area (20%, 60%, and 100%). Triangular pin geometry, different feed rates (20, 40, and 60 mm/min), and different tool rotation speeds (1250, 1750, and 2250 rpm) were used as FSW process parameters. Tensile testing was performed to determine weld strength and hardness measurements, and visual inspections were performed. Color measurements were made on the test samples before and after the welding process, and the relationship between welding performance and color was evaluated. The best welding strength was obtained as 17.83 ± 0.68 MPa at a feed rate of 20 mm/min, a tool rotation speed of 1750 rpm, and a part with a 60% infill ratio in the welding zone. In the sample with the best weld strength, the temperature was measured as 198.97 °C. Color changes in the weld area of parts with 60% and 100% infill ratios were measured between 78.9–82.2 and 79.1–84.5, respectively. It was determined that the color change decreases as the weld strength increases in these parts. The results show that with the proposed new part design, the FSW method can be used at low infill ratios, and the weld strength can be evaluated based on the color changes in the weld zone.

## 1. Introduction

Additive manufacturing (AM) is one of the recent advancements in manufacturing technologies, where the transition from design to product manufacturing is rapid compared to traditional manufacturing practices. In FDM, one of the additive manufacturing methods, the designed part is added layer by layer and takes its final shape. Various polymer-based materials are used in the FDM method. Among different polymer materials, polylactic acid (PLA), which is obtained from renewable resources and has advantages such as environmental friendliness and ease of printing, is among the most used filaments [1]. While 3D printing is a rapid and effective method for producing complex geometries, the size of the product to be printed is limited by the printer’s bed size. In order to obtain large-volume parts, assembling 3D-printed products or combining them with a method is required. Two different methods are used in joining plastics: permanent joining and semi-permanent joining. The friction stir welding (FSW) method, one of the permanently joining methods, is a solid-state welding method known for its ability to weld parts that are difficult to join and to be applied to different material pairs [2,3,4,5,6]. Combining FSW with 3D-printed products has attracted the attention of researchers, and although the studies are limited, they have made significant contributions to the literature [7]. In a study in which polymer materials were joined using FSW, it was reported that the mechanical performance of the welded material increased compared to the unwelded material and that among the pin diameter and geometry, feed rate and tool speed, shoulder diameter and geometry, and tool tilt angle parameters used in the experiment, the most effective parameter on the joining was the feed rate [8].

In another study on acrylonitrile butadiene styrene (ABS) plates, the feed rate (20, 40, and 80 mm/min), tool speed (800, 1250, and 1600 rpm) and material preheating temperature (50, 80, and 100 °C) parameters were changed, and their effects on the weld quality were examined. The tensile test gave the best results when the feed rate was selected as the lowest and the tool rotation speed and temperature selected as the highest [9]. Tiwary et al. [10], who conducted research on the joining of ABS-ABS, ABS-PLA, and PLA-PLA sheets by the FSW method, investigated the role of nylon microparticles (wt. 4%, 8%, and 12%), which they added to the welding zone at different rates, in the FSW process. In experiments conducted at different tool speeds (1000, 1100, and 1200 rpm) and feed rates (25, 30, and 35 mm/min), 76% of the main material strength was obtained, although nylon microparticles showed their effect on PLA-PLA joining. In another study examining the joining ability of HDPE, PVC, and PA6 thermoplastics using the FSW method, the most effective joint compared to the base material was obtained with 70% efficiency for HDPE [11]. Derazkola et al., who studied the joining of PC sheets, examined the effect of FSW process parameters on mechanical properties. The results showed that when the tool rotation speed was 2200 rpm, the feed rate was 105 mm/min, the tilt angle was 2.5°, and the plunge depth was 1.2 mm, the best strength value was obtained with an efficiency of approximately 82% compared to the base material [12]. Moochani et al. investigated the impact of FSW process parameters on the mechanical properties of polypropylene (PP) sheets. In the test conducted at different tool rotation speeds (360, 565, and 950 rpm), feed rates (24, 40, and 60 mm/min) and temperatures (130, 150, and 170 °C), they obtained a welded material with 96% efficiency in tensile strength and 98% efficiency in elongation value. Additionally, it was reported that the most effective parameter affecting the tensile strength of the welded sample was the tool temperature, and the most effective parameter affecting the elongation value was the rotation speed [13]. Koçar et al. investigated the effects of FSW process parameters on the joining of PLA wood material with PLA-CF and PLA Plus materials. Three different pin profiles (triangle, square, and screw), tool rotation speeds (1250, 1750, and 2250 rpm), and feed rates (20, 40, and 60 mm/min) were used in the study. When the processing parameters were selected as square pin geometry, 20 mm/min feed rate, and 1750 rpm tool rotation speed, PLA wood showed the highest welding strength with 74.5% efficiency. The best welding strengths were obtained with the same process parameters when combining PLA wood plates with PLA-CF and PLA Plus materials [14]. Another study examined the effect of infill ratio on weld strength values when combining PLA Plus sheets printed at different infill ratios (20%, 40%, 60%, 80%, and 100%). Tensile tests and temperature measurements were conducted to examine the effects of FSW process parameters (feed rate: 50 mm/min and 100 mm/min; rotation speed: 1000 and 1500 rpm) on the weld structure and mechanical properties. The highest welding strength compared to the base sheet material was achieved with 112.38% efficiency at an 80% infill ratio [15]. They also stated that surface tunnel defects occurred due to a lack of material at 20% and 40% infill ratios.

Additionally, apart from 3D-printing parameters, filament color is another parameter affecting the mechanical properties of the parts [16,17]. Coloring the filament is achieved by adding masterbatch dye, which can be in the desired colors and the form of granules, into PLA granules, for example, during the production stage. Masterbatch is used in the production process at a rate of 4% by weight for PLA and 2% by weight for ABS to create the main mixture. Masterbatch (color granules) added to the base material to give color affects the mechanical properties of the base material. The study by Frunzaverde et al. showed that PLA filament color is an effective parameter of tensile strength. The natural- (no mast added), black-, red-, and gray-colored filaments used in the study were produced in different layer thicknesses (0.05, 0.10, 0.15, and 0.20 mm). In all layer thicknesses, the highest tensile strength value was obtained by the gray color (57.10–59.82 MPa), while the lowest strength was obtained by the black color [18].

In their study, Wittbrodt and Pearce used five different colors and obtained the highest tensile strength in the natural color [19]. Pandžić et al. used 14 different-colored PLA filaments to determine the filament color effect. While the tensile strength varied between 35–46 MPa depending on the color scale, the best values were seen in red, black, and gray filaments. The study also reported a 300% change in toughness value and approximately 400% change in strain value depending on color [20]. Another study stated that according to color theory, all colors were obtained by mixing primary colors and that the mechanical properties of colored filaments varied depending on the color pigments. The mechanical properties of filaments in seven different colors were examined to determine whether the mechanical properties of the filaments and color theory were compatible. Three of these (red, yellow, and blue) were primary colors, and the other colors (orange, purple, green, and black) were the colors obtained from mixing primary colors. As a result, it was found that the mechanical properties of filaments in other colors can be evaluated by taking the mechanical properties of the filaments in the primary colors as a reference. The best result for tensile strength was obtained by the purple color; in compressive stress, it was obtained by the orange color; and for the flexural test, it was obtained by the purple color. As a limiting factor in the study, it was stated that the manufacturing companies kept the color pigment contents secret. Since the color spectrum was wide, it was difficult to determine the color, especially in filaments consisting of two primary colors [21].

Industrial products do not consist only of single-colored parts. Products that use different-colored materials together may be preferred by consumers simply because of their visual appeal. However, there are some difficulties in combining colored plastic materials. For example, it is difficult to weld colored materials using laser welding. Although laser welding such as FSW is preferred to reduce the part’s weight, the material’s permeability to be welded is important in laser welding. When colors or pigments are added to thermoplastics, the permeability properties of these materials will change. Depending on the color additive used and the resulting color combination, the performance of the laser welding process is affected [22]. In such cases, the color component must be considered in the welding process. FSW is an effective method for joining different types of materials. It is not selective concerning color. Many methods are used for welding plastics. One of these methods is ultrasonic plastic welding. However, the method can be used to join two different thermoplastic materials to a limited extent. Each method has its advantages and disadvantages. For these reasons, the FSW method was preferred to determine the relationship between color change and strength more clearly using materials of different colors.

When the literature was examined, it was determined that the joinability of parts (similar or dissimilar material pairs) produced with 3D printers using FSW and the effects of FSW process parameters on weld quality should be investigated. However, one of the most important advantages of 3D printers is the ability to print parts at different infill ratios. In the FSW process, the joining process is carried out by providing material flow with tools with different pin profiles. The void areas with low infill ratios obtained by 3D printers negatively affect the heat generation and material flow and therefore the joining process during FSW. Therefore, there is a need for innovative approaches to joining low-infill parts with FSW. Based on this feature, the authors previously studied the weldability of parts printed at different infill ratios using FSW. Their study evaluated the weld strength of parts with low infill ratios.

In the study, it was noted that high levels of welding defects were observed when joining parts with 20% and 40% infill ratios. In contrast, parts with a 60% infill ratio could only be joined with specific process parameters [15]. As a result of the study, difficulties in welding parts with low infill ratios were recognized. This present study is proposed as a solution to change the part design to combine parts with different infill ratios. In addition to this purpose, it was also discussed whether color changes in welded materials are related to weld strength.

First, PLA Plus sheets in two different colors (black and orange) with a 20% infill ratio were produced with the 3D printer. All these parts have a 20% infill ratio. Based on the proposed approach, the parts’ infill ratio of the weld zone were printed at different ratios (20%, 60%, and 100%). The FSW method was used to join the produced sheets. As FSW process parameters, three different tool rotation speeds (1250, 1750, and 2250 rpm) and three different tool feed rates (20, 40, and 60 mm/min) were preferred. Tensile, hardness, and impact notch tests were then applied to determine the weld strength and hardness change. Additionally, thermal images were taken during FSW to interpret the weld strength. The color change in the welding zone and its relationship with the welding quality were examined. No study has been found in the literature in which the difference between the color formed in the welding zone and the colors of the base materials being welded is associated with the weld strength. This study evaluated the welding process performance of parts produced with a 3D printer based on color variations. The aim is to provide reference for researchers working on welding colored, 3D-printed materials.

## 2. Material Method

### 2.1. Experiment Materials

The experiments used PLA Plus material in two different colors (orange and black) as filament material. The mechanical properties of PLA Plus filament are given in Table 1. Since PLA is produced from organic materials (edible sources such as corn starch, cassava, sugar beet, and sugar cane), it is harmless to human health, non-toxic, and biodegradable [23,24,25]. In addition, PLA material is the most commonly used filament material in fused deposition modeling (FDM) due to its ease of printing and production. There are also derivatives with improved mechanical properties that can be added by additional additives (PLA Plus, PLA tough, PLA wood, PLA CF, etc.). PLA Plus is a filament with increased toughness and impact resistance obtained by adding 2% calcium carbonate into PLA.

### 2.2. Printing Samples from 3D Printer

**Figure 1:**
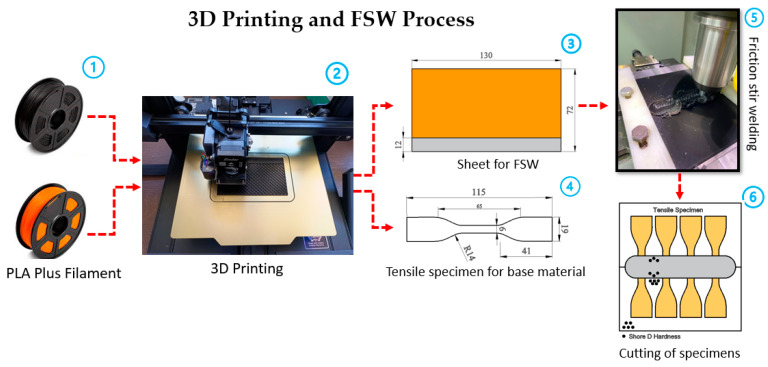
Three-dimensional printing and FSW process: (1) PLA Plus filament; (2) Creality Ender 3S-1 printer; (3) sheet for FSW; (4) tensile specimen; (5) FSW process; (6) sample preparation after welding.

In the experiments, PLA Plus (Figure 1(1)) filaments were printed using the Creality Ender 3-S1 printer with the fused deposition modeling technique (Figure 1(2)). A tensile sample was prepared with a 3D printer to determine 130 × 72 × 5 mm plates (for FSW) and base material properties (yield, UTS, elongation, and hardness) (Figure 1(3 and 4)). Tensile samples were printed at 20%, 60%, and 100% infill ratios by ASTM D638-10 [27] standards. Three-dimensional printing parameters (printing and bed temperature, infill ratio, printing direction, etc.) significantly impact the part’s mechanical properties. The 3D printing parameters include the following: the printing temperature was 208 °C, build plate temperature was 60 °C, and the print speed was 60 mm/s, and it was printed flat in the XYZ axis layout. Samples with 20% and 60% infill ratios were produced in a grid pattern, and the samples with 100% infill ratios were produced by following a linear pattern with an angle of 45/−45 degrees in each layer, respectively.

The plates printed on a 3D printer were combined with the FSW method (Figure 1(5)). Finally, to determine the weld strength, tensile samples were removed from the welded samples, and hardness measurements were taken from three different regions (base material, heat-affected zone, and welding zone) (Figure 1(6)). To determine the weld strength and base material properties, tensile tests were carried out on a WDW-5 model tensile device with a capacity of 5 kN, at a tensile speed of 2 mm/min and room temperature (21 °C).

A LOYKA D-type Shore hardness durometer was used for hardness measurements of welded specimens, and measurements were performed according to ASTM D2240 standard. Hardness measurements were performed in five repetitions from the base material, and HAZ area and averages were taken.

The temperature generated during FSW was measured using a Fluke Ti-32 infrared thermal imager with a temperature range (−20–600 °C) and an accuracy of ±2 °C. The distance to the welding zone was fixed at 75 ± 5 cm. The angle of the camera lens was fixed at 45 ± 5° from the welding surface. Thermal images were taken every 30 s. The ones with the highest temperature values were selected from the thermal images.

### 2.3. Experimental Strategy and 3D Part Design

With the increasing use of 3D printers in recent years, the need to combine parts produced in 3D printing has emerged. Semi-permanent (mechanical connections) and permanent (gluing and welded joining) joining methods are used in plastic materials. In this study, the solid-state welding FSW method, one of the permanent joining methods, was used to join the materials. FSW is mainly applied to join materials that are difficult to weld by fusion welding, such as aluminum and titanium alloys [28]. In addition, the FSW method is used to join polymer matrix composites. AA6082-T6-PP [29], ABS-PA6 [30], HDPE-PVC-PA6 [11], PLA wood [14], PLA [31,32], HDPE [33], PP/PC [34], wood/plastic [35], and Nylon 6 [36] materials are as examples of materials used for joining polymer material pairs. The process parameters used in the FSW method significantly affect the weld quality. To determine the effect of process parameters on welding quality, various studies have been carried out on feed rate [37], tool rotation speed [38], shoulder [39], pin geometry [40], tilt angle [41], and force [42].

**Figure 2:**
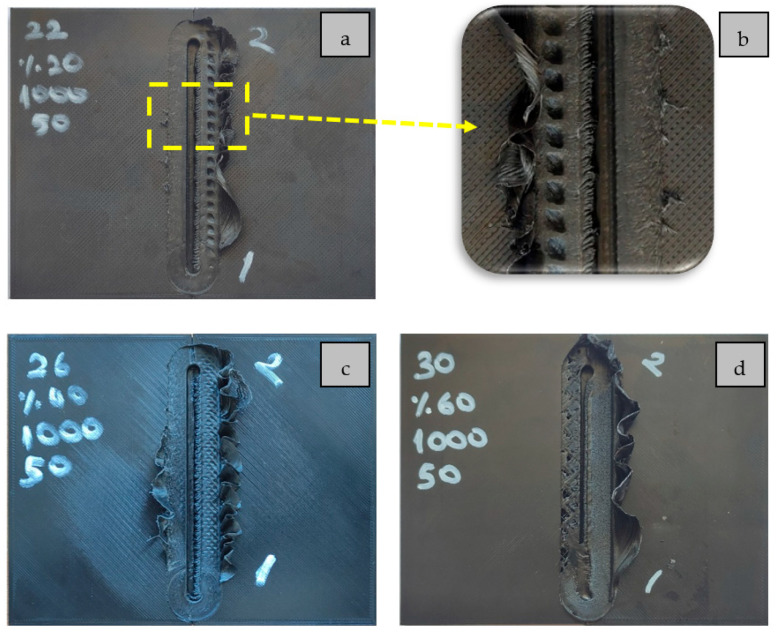
Post-FSW image of materials with different infill ratios: (**a**) 20%, (**b**) 20% surface tunnel defect, (**c**) 40%, and (**d**) 60% [15].

In the studies conducted on joining the materials produced with the FDM technique (3D printers) by FSW, it was seen that the infill ratio of the materials was selected as 100%. However, one of the most important advantages of 3D printers is the ability to produce parts at a low infill ratio. However, the FSW method makes parts produced at low infill ratios difficult to join [15]. Due to the nature of FSW (in parts with 100% infill ratio), the heat generated by the contact of the tool shoulder with the part plasticizes the material in the welding zone. The plasticized material is carried to the other side (from AS to RS) with the mixing tip, and the materials are mixed. However, the necessary conditions for joining are not met in parts with a low infill ratio because the contact (friction) of the tool shoulder with the part will be low due to the voids. Using the FSW method, Anaç et al. tried joining PLA Plus plates with different infill ratios (20, 40, 60, 80, and 100%). They stated that the weld strength improved at a relatively high feed rate at an 80% infill ratio. They observed that at other infill ratios (20%, 40%, and 60%), surface tunnel defects occurred in the parts, negatively affecting the weld strength. From the experiments they conducted in their study, the post-welding images of PLA Plus plates with 20% (Figure 2a,b), 40% (Figure 2c), and 60% (Figure 2d) infill ratios using a feed rate of 50 mm/min and a tool rotation speed of 1000 rpm are given in Figure 2. Due to the low infill ratio, a surface tunnel defect occurred along the weld during FSW.

**Figure 3:**
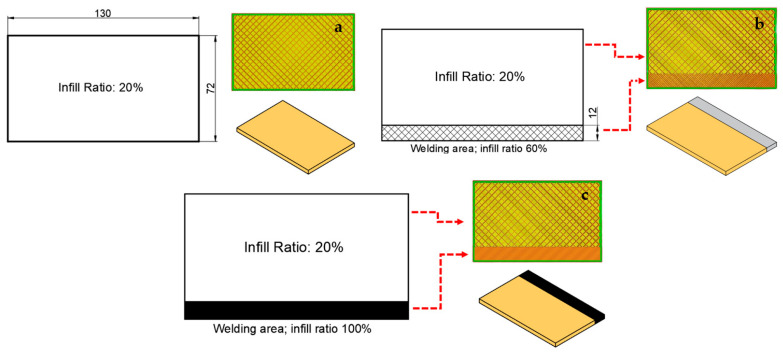
View of the parts designed for joining parts with different infill ratios using FSW: (**a**) 20% infill ratio in the weld zone, (**b**) 20% infill ratio in the weld zone, and (**c**) 100% infill ratio in the weld zone.

Figure 3 shows parts with different infill ratios in the weld zone. In Figure 3a, a conventional plate design (reference sample) with 20% infill ratio and dimensions of 130 × 72 × 5 mm; in Figure 3b, a plate design with a 12 mm weld zone width and 60% infill ratio; and in Figure 3c, a plate design with a 12 mm weld zone width and 100% infill ratio are shown. Weld zone infill ratio and FSW process parameters are given in Table 2. The experiments were carried out under the conditions of 20% infill ratio for the parts; 20%, 60%, and 100% infill ratios in the welding zone; at 20, 40, and 60 mm/min tool feed rate; and at 1250, 1750, and 2250 rpm tool rotation speed.

### 2.4. Determining FSW Parameters (Welding Speed, Rotational Speed, and Pin Geometry)

FSW, a solid-state welding technology, joins two materials by mixing them along the weld joint. FSW technology consists of a tool consisting of a shoulder and pin profile, a fixture used to clamp the materials so they do not move, and a machine to provide rotational/advancing movements for the tool. During FSW, the tool shoulder friction with the material pair generates heat in the weld zone. With the heat generated in the weld zone, the material pair flows and plasticizes. Meanwhile, the tool rotation mixes the materials, and with the tool feed, the mixing process is repeated along the weld line, and the welded joint is formed. This results in severe plastic deformation and material flow in the weld zone. FSW is environmentally friendly, does not require additional metals, and can join dissimilar materials, resulting in better mechanical properties, lower residual stress and deformation, and fewer weld defects.

In the FSW method, process parameters directly affect the weld quality. Process parameters must be determined again for each pair of materials. In sectoral applications, FSW process parameters are determined according to the operator’s experience or trial-and-error method. While this increases the amount of scrap, it causes a loss of time and increased costs.

**Figure 4:**
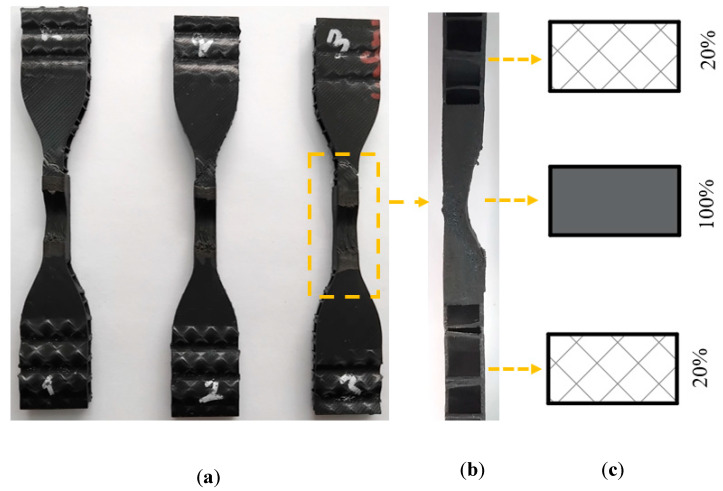
After the preliminary experiment. (**a**) A view of the tensile samples; (**b**) a side view of the welding zone; (**c**) part and weld zone infill ratio (triangle pin geometry, feed rate 40 mm/min, and tool rotation speed 1250 rpm).

According to the literature, FSW process parameters in joining plastics include a range of 3–60 mm/min for tool rotation speed in studies on PLA (3D-printed), while for other plastic materials (Table 3), the feed rate is between 2–115 mm/min, and the tool feed rate is between 600 and 3000 rpm. Accordingly, it can be said that FSW parameters should be changed according to the material production method and material properties. Based on literature studies, preliminary experiments were conducted with three different pin geometries (triangle, square, and screw), two feed rates (20 and 40 mm/min), and two sets of feed rates (1250 and 2250 rpm) (Table 3). The parameters used in preliminary experiments were determined as PLA Plus (black) filament, 100% infill ratio for the welding area, and 20% infill ratio for the entire part. Figure 4a shows the tensile samples after the preliminary test, Figure 4b shows the side view of the tensile sample, and Figure 4c shows the representative view of the infill ratio. The infill ratio of the welding zone (100%) and the base material (20%) can be seen.

The preliminary test results are given in Table 4. The highest weld strength was obtained as 7.6 MPa under the conditions of triangle pin geometry, at 40 mm/min feed rate, and 1250 rpm tool rotation speed. According to these results, a triangular tip was used as the pin geometry. Anaç [51] stated in her study on joining HDPE and PLA Plus plates that the most practical geometry was the triangular pin geometry. Other parameters were determined as 20, 40, and 60 mm/min feed rate and 1250, 1750, and 2250 rpm tool rotation speed.

**Figure 5:**
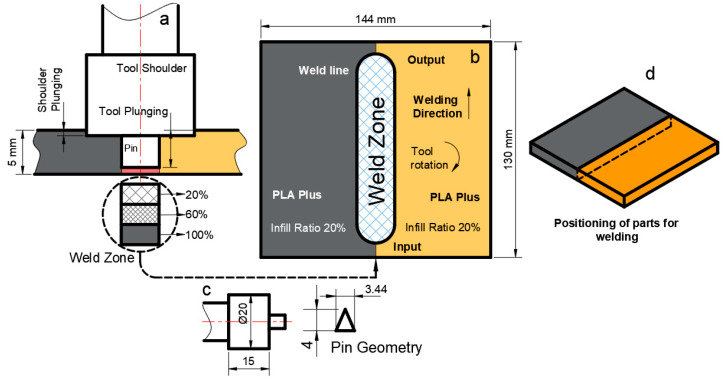
FSW constant process parameters and weld zone. (**a**) Tool shoulder penetration depth and part infill ratios, (**b**) FSW weld characteristics, (**c**) pin geometry and tool characteristics, and (**d**) positioning of parts for welding.

The FSW process has many process parameters. Process parameters other than the parameters mentioned above (feed rate and rotational speed) are given in Figure 5. Tool plunging was 4.5 mm, tool shoulder diameter was 20 mm, and shoulder plunging was 1 mm (Figure 5a). The tool entry/exit points, welding direction, and tool rotation direction are given in Figure 5b, and the dimensions of the pin geometry and tool characteristics are given in Figure 5c, while the positioning of parts for welding is shown in Figure 5d.

### 2.5. Measurement of Color

Color measurements of the samples printed on a 3D printer and welded together were made using the CIE L*a*b* color system on the CHN-SPEC CS-410 portable spectrometer device. In the CIE color system, the values in which color is expressed are determined according to three color coordinates: “L*, a*, b*”. L* (darkness-lightness); a* (greenness-redness); and b* (blueness-yellowness) parameters of the test parts were measured from samples printed at 20%, 60%, and 100% infill ratios before welding and from the welding zone after welding. Each measurement was taken in five repetitions, and the colors of the samples were determined based on the average of the five measurements. Reference color measurement values were taken from 3D-printed parts that were not welded. Other color measurements were made along the weld line after joining with FSW (before the 3D-printed parts were cut). To express the color difference of two parts with a single value, Δa*, Δb*, ΔL*, and ΔE* values were calculated using the relevant formulas [52,53].

## 3. Results and Discussion

### 3.1. Base Materials Properties

**Figure 6:**
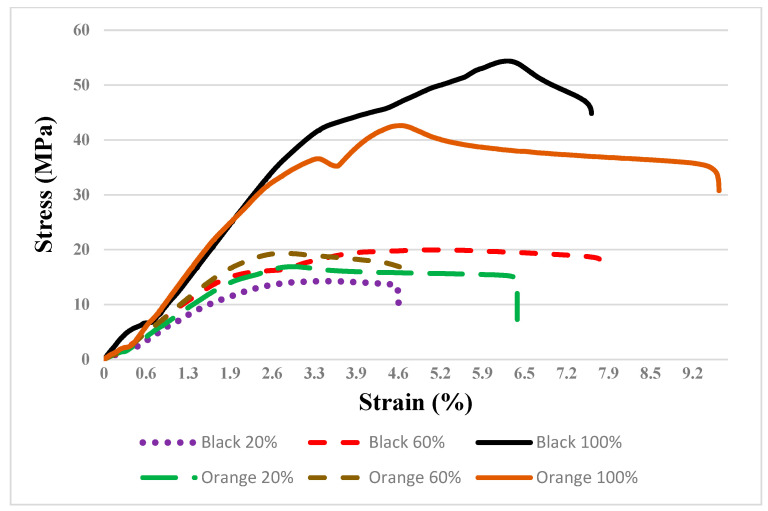
Stress and strain curves for PLA Plus (infill ratios of 20%, 60%, and 100%).

The tensile graphs (stress–strain) of PLA Plus filament at different infill ratios are provided in Figure 6. As a result of the tensile test, it was determined that the mechanical properties varied depending on the color pigment. In their study on the effect of color pigments on mechanical properties, Gao et al. indicated that the tensile strength of PLA varied between 39.9 MPa and 52.5 MPa, the compressive strength between 48.2 MPa and 62.0 MPa, and the flexural strength between 52.5 MPa and 65.9 MPa, depending on the color [21]. In the study, the tensile strengths (black and orange parts, respectively) were determined as 53.07 MPa and 42.32 MPa for a 100% infill ratio, 19.61 MPa and 19.26 MPa for a 60% infill ratio, and 14.35 MPa and 16.80 MPa for a 20% infill ratio. The difference in UTS according to color was observed most prominently at the 100% infill ratio. Additionally, as the infill ratio decreased, the mechanical properties of the orange filament (16.8 MPa) relatively exceeded those of the black filament (14.35 MPa). It can be said that this situation arises from the different effects of color pigments on mechanical properties depending on the infill ratio. Different filament colors occur due to color additives added to the filaments [21]. However, color reproduction in 3D printing depends not only on the coloring matter. All other materials involved in the part’s manufacturing process will affect the resulting color [54,55]. “Additives” added to the polymer can change thermal properties such as crystallization [19] and viscosity [16,54], adhesion between layers, surface properties [ATIF 3], and mechanical properties of the part [56,57]. Also, adding black and orange color additives to the filament material will result in differences in strength and other properties. In addition, the viscosity of filament materials varies depending on the amount of pigment. Pigment lack causes low melt viscosity. In this case, a smooth and homogeneous part surface is formed in printing [16,58]. High viscosity, especially at low infill ratios, causes problems in the adhesion and bonding of the layers. Since the flow is difficult, the layers cool down before fully fusing together. In a 100% infill part, the temperature is maintained within the layers, while the hollow structure is more prone to cooling. It is difficult to fully explain this phenomenon, as the amounts of colorants added to commercial filaments are unknown. However, due to these differences, it is thought that the ability of the layers to adhere to each other is reduced in the black 3D part printed at a 20% infill ratio compared to the orange color.

Shore D hardness values according to the infill ratio are provided in Table 5. Accordingly, there was an increase in the hardness value as the infill ratio increased. If hardness is defined as resistance to sinking, the resistance to sinking decreases due to the voids formed in the part as the infill ratio of the part decreases. Additionally, no significant difference was observed in hardness changes according to color.

### 3.2. Visual Inspection after FSW

**Figure 7:**
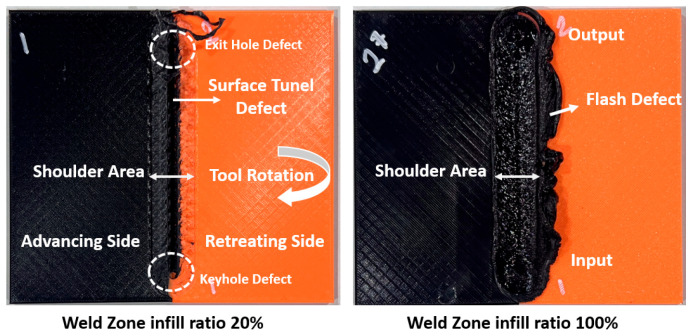
View of the parts with 20% and 100% infill ratios in the weld zone after joining.

In the FSW process, the tool approached the part from above with a feed rate of 20 mm/min. To generate the required heat for welding, the tool was kept at the entry point for 10 s and advanced 100 mm to the exit point at different feed rates. Figure 7 shows two welded specimens with 20% and 100% infill ratios in the weld zone. Surface tunnel, keyhole, and exit hole defects were determined in samples with a 20% infill ratio in the weld zone. It can be said that this situation is due to insufficient material in the shoulder friction area and mixing area. Flash defect formation was determined in the sample with a 100% infill ratio in the weld zone. This situation occurs when the material in the weld zone flows out due to excessive plasticization. Figure 7 provides the fundamental concepts of the FSW process (entry/exit points, advancing side (AS), retreating side (RS), and tool rotation direction).

**Figure 8:**
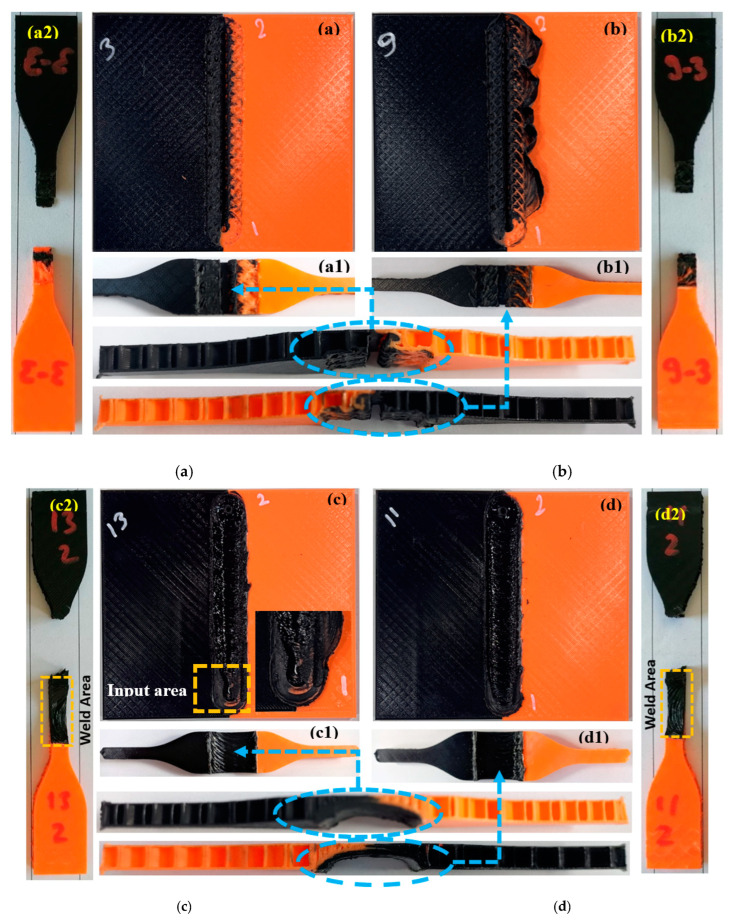

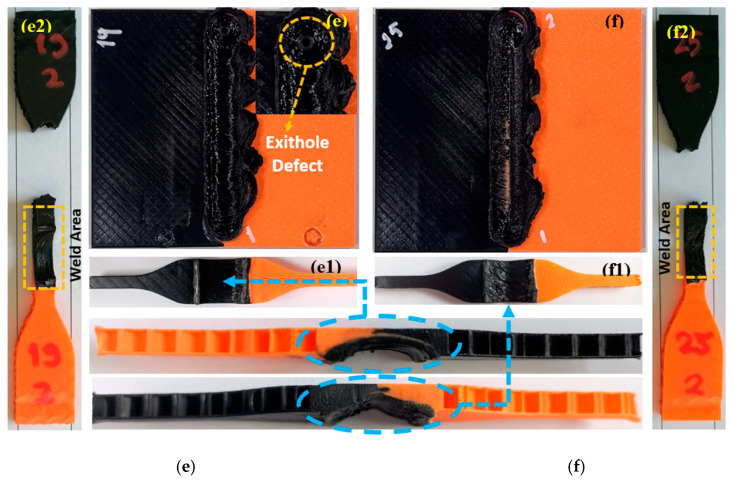
Sample images with the best and worst weld strength according to the weld zone infill ratio. (**a**) Minimum UTS for 20% infill ratio. WA: 20% infill ratio, FR: 20 mm/min, RS: 2250 rpm (1.66 MPa) (Experimental number: 3). (**b**) Highest UTS for 20% infill ratio. WA: 20% infill ratio, FR: 60 mm/min, 2250 rpm (3.32 MPa) (Experimental number: 9). (**c**) Minimum UTS for 60% infill ratio. WA: 60% infill ratio, FR: 40 mm/min, RS: 1250 rpm (13.33 MPa) (Experimental number: 13). (**d**) Highest UTS for 60% infill ratio. WA: 60% infill ratio, FR: 20 mm/min, RS: 1750 rpm (17.83 MPa) (Experimental number: 11). (**e**) Minimum UTS for 100% infill ratio. WA: 100% infill ratio, FR: 20 mm/min, RS: 1250 rpm (11.07 MPa) (Experimental number: 19). (**f**) Highest UTS for 100% infill ratio. WA: welding area; FR: feed rate; RS: rotational speed. WA: 100% infill ratio, FR: 60 mm/min, RS:1250 rpm (16.82 MPa) (Experimental number: 25).

Figure 8 shows the visual examination of the experiments with 20%, 60%, and 100% infill ratios in the weld zone and the highest and lowest weld strengths obtained. In Figure 8a,b (samples with a 20% infill ratio), it was determined that a surface tunnel defect occurred due to lack of material (Figure 8a,b). Additionally, flash defects can be seen in Figure 8b. When Figure 8(a1,b1) are examined, it is seen that the two plates were better mixed in Figure 8(b1), and the voids of the orange plate were filled with black material. Good material flow ensured a better joining of the two plates and increased the welding strength. When the post-weld tensile samples in Figure 8(a2,b2) are examined, it is seen that the fractures occurred in the weld zone. This situation shows that the weld strength is below the desired level. This study aims to increase the weld strength by increasing the infill ratio in the weld zone of parts with a low infill ratio.

In Figure 8c,d, images of samples with a 60% infill ratio in the weld zone are given. At the 60% infill ratio, there was an improvement in the weld zone compared to the 20% infill ratio. The welding strength also varies depending on the process parameters (feed rate and tool rotation speed). When Figure 8c,d are compared, it is seen that the weld lines formed depending on the tool rotation direction formed more smoothly in Figure 8d. This shows that the FSW process parameters were appropriate, and the material flow occurred more smoothly. In Figure 8c, a welding defect occurred at the tool entry point. It can be said that this situation arises from the insufficient plasticization of the material due to the low tool rotation speed and relatively high feed rate. Inappropriate process parameters lead to insufficient heat generation, inadequate plasticization of the material in the weld zone, and imbalanced material flow around the pin [59]. Additionally, exit hole defects were observed at the tool exit point. Keyhole and exit hole defects are among the most common defects encountered in the FSW process [60]. It is observed in Figure 8(d1) that although there are voids in the weld zone, they are relatively reduced compared to Figure 8(c1). When examining the tensile samples in Figure 8(c2,d2) after welding, it can be seen that a rupture occurred outside the weld zone (from the zone with a 20% infill ratio). Depending on the color pigment, the tensile strength (UTS) was determined to be between 14.35–16.80 MPa for samples with a 20% infill ratio and 19–20 MPa for samples with a 60% infill ratio (Table 4). It was determined that the samples’ ultimate tensile strength (UTS) in the weld zone with the 60% infill ratio varies between 13.33–17.83 MPa. The weld strength falls within the range of the ultimate tensile strength (UTS) values determined for the base material at both 20% and 60% infill ratios.

Figure 8e,f show visuals of samples of weld zones with a 100% infill ratio. At the 100% infill ratio, there was an improvement in the weld zone compared to the 20% infill ratio. It was determined that the post-FSW weld strength of the parts with the 100% infill ratio in the weld zone was between 11.07–16.82 MPa. In Figure 8(e1,f1), it can be seen that the weld lines are continuous. This shows that the material flow is smooth, and there is a more balanced mixing. Exit holes and flash defects were determined in all samples. When Figure 8(e2,f2) post-welding tensile samples are examined, it is seen that the fractures occurred outside the weld zone (welding zone with the 20% infill zone).

### 3.3. Results of Weld Strength after FSW

Table 6 shows the tensile strengths of PLA Plus plates welded using three different feed rates and three different tool rotation speeds at 20%, 60%, and 100% infill ratios of the weld zone. In addition, welding efficiency (%) was determined for each experiment, taking black and orange colors as a reference. Significant welding defects occurred in the samples with a 20% infill ratio in the weld zone, and weld strength was obtained between 1.66–3.32 MPa. In the samples with 60% and 100% infill ratios of the weld zone, the highest weld strength was obtained as 17.83 and 16.82 MPa, respectively, and the weld efficiency was 124.25%/106.13% and 117.21%/100.12% compared to the fundamental mechanical properties of black and orange filaments.

**Figure 9:**
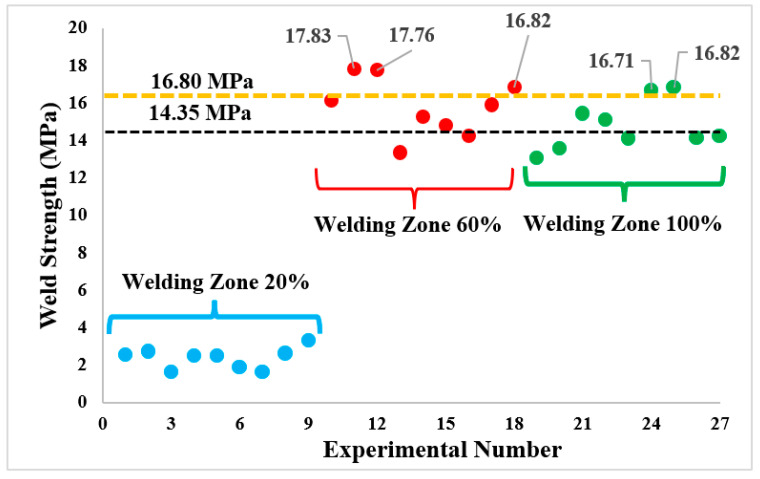
Distribution of weld strengths after FSW.

For a general evaluation, the change of UTS according to the weld zone infill ratio is given in Figure 9. In Figure 9, the horizontal black line represents the strength of the filament with black color pigment at the 20% infill ratio, and the orange line represents the filament with orange color pigment. Significant weld errors occurred in the samples with the 20% infill ratio in the weld zone due to a lack of material, and the weld strength remained below the desired level. The weld strength increased significantly at 60% and 100% infill ratios of the weld zone. The welding performance achieved at the 60% infill ratio of the weld zone was better than that at the 100% infill ratio. The highest welding strength (17.83 ± 0.68 MPa) was obtained at a 60% infill ratio, tool rotation speed of 1750 rpm, and 20 mm/min feed rate. At the 100% infill ratio, the tensile strength of the base materials according to colors was found to be 53.07 (black) and 42.32 MPa (orange). However, contrary to expectations, the weld strength was higher in parts with the 60% infill ratio compared to the 100% infill ratio. It can be said that this is due to the difference between the mechanical properties of the welding area and the base material. It is thought that the 100% infill ratio of the weld zone causes the stress to accumulate in the base material during tension and causes early crack initiation. When comparing the tensile results of the base material with the weld strengths in Table 5, it is observed that the weld strengths are close to the tensile results of specimens with a 20% infill ratio. This is because the strength in the weld zone of samples welded with 60% and 100% infill ratios is limited by the tensile strength of the base material.

### 3.4. Weld Strength According to FSW Process Parameters

**Figure 10:**
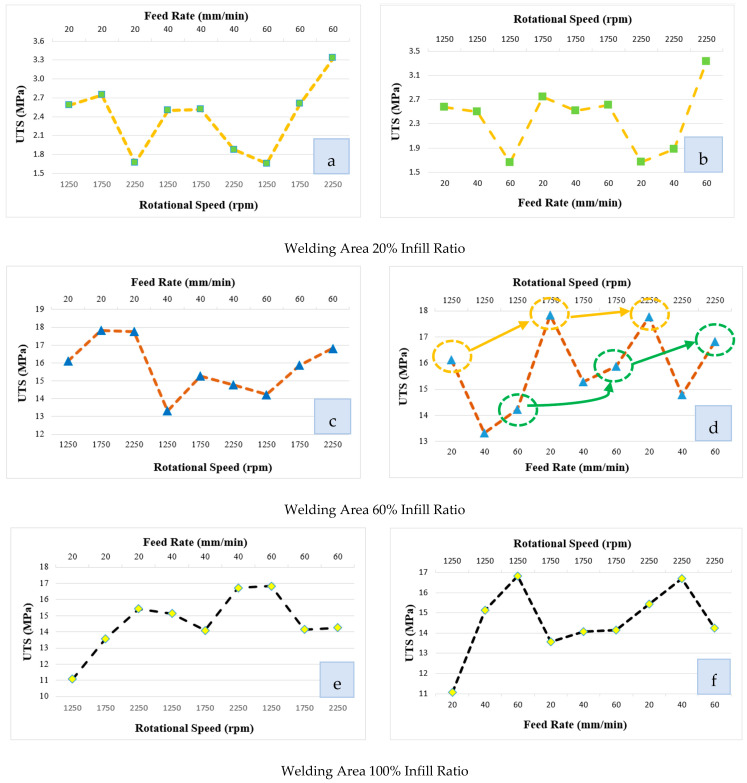
Change of weld strength according to tool rotation speed and feed rate. (**a**,**b**) Welding area at 20% infill ratio. (**c**,**d**) Welding area at 60% infill ratio. (**e**,**f**) Welding area at 100% infill ratio.

Figure 10 shows the variation of weld strength according to rotational speed and feed rate for all infill ratios. Since welding errors occurred due to lack of material at the 20% infill ratio, the effect of the process parameters was low (Figure 10a,b). It was determined that when the feed rate was 60 mm/min, the weld strength improved as the tool feed rate increased. Similarly, when the tool rotation speed was 2250 rpm, the weld strength improved as the feed rate increased.

In Figure 10c,d, the change in welding strength of the parts with a 60% infill ratio in the welding zone according to the tool rotation speed and feed rate is given. In FSW, the generation of heat required for welding plays a key role in the success of the weld. Excessive heat generation caused by friction leads to over-plasticization in the weld zone and material flowing out of the weld zone. However, insufficient heat generation during FSW leads to inadequate material flow. Understanding the relationship between process parameters and weld quality is crucial for achieving the desired weld quality in FSW [61,62,63].

In the weld zone with a 60% infill ratio, the best weld quality was determined to be 17.73 MPa at 20 mm/min feed rate and 1750 rpm tool rotation speed. In Figure 10c, when the tool rotation speed increased from 1250 rpm to 1750 rpm at a 20 mm/min feed rate, the weld quality improved, while there was no change at 2250 rpm. When the feed rate was 60 mm/min, welding strength increased with the tool rotation speed. When Figure 10d is examined, it is seen that increasing the rotation speed at 20 and 60 mm/min feed rates positively affected the weld strength. Welding strength was relatively high at the 20 mm/min feed rate. The reason for this is that due to the voids in the weld zone, the friction between the tool shoulder and the material must be high to obtain the necessary heat. Therefore, as the rotation speed increases, the friction between the tool shoulder and the part increases, and the heat also increases as a result. By increasing the feed rate by 60 mm/min, local friction and resulting heat decreased. This situation negatively affected the material flow and reduced the welding quality.

In Figure 10e,f, the change in the welding strength of the parts with a 100% infill ratio in the welding zone according to the tool rotation speed and feed rate is given. When Figure 10e is examined, it can be seen that the best welding strength was obtained as 16.82 MPa under a tool rotation speed of 1250 rpm and feed rate of 60 mm/min. While the weld strength improved as the tool rotation speed increased at a low tool feed rate (20 mm/min), it decreased at a 40 mm/min feed rate and 1750 rpm rotational speed and increased at 2250 rpm rotational speed. The 60 mm/min feed rate started to decrease as the tool rotation speed increased. In their study, Anaç [15] et al. stated that as the infill ratio increases, the weld strength increases. In this study, the weld strength varied depending on the mechanical properties of the weld with the lowest infill ratio due to the regional variation in the infill ratio of the parts because in parts with 60% and 100% infill ratios, fracture occurred from the zone with a 20% infill ratio before cracking or fracture occurred in the weld zone.

### 3.5. Weld Strength and Color Difference after FSW

This study carried out color measurements on all samples (from base materials and weld zones). The results of the color measurements are given in Table 7.

**Figure 11:**
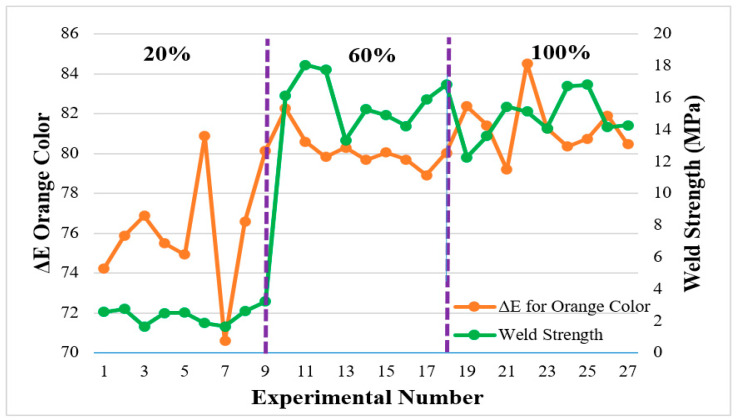
Relationship between weld zone color change and weld strength in orange part.

**Figure 12:**
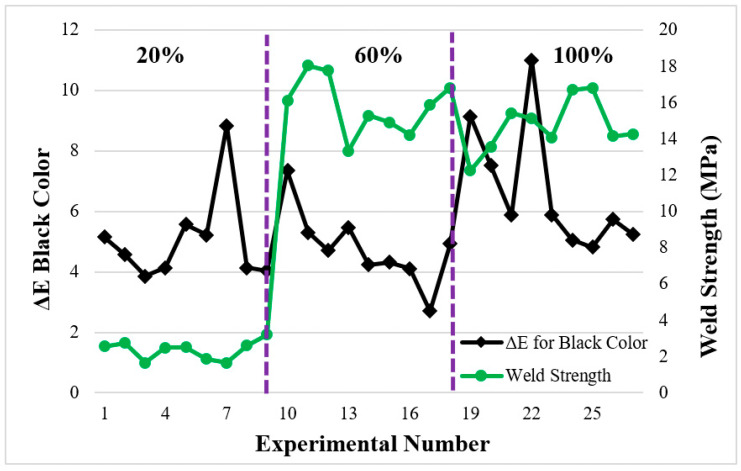
Relationship between weld zone color change and weld strength in a black part.

In Figure 11 and Figure 12, the difference between the color formed in the weld zone and the base colors is given, and the relationship of these values with the weld strength is shown. Color measurements of parts with different infill ratios in the welding zone were taken after welding. Figure 11 shows that the orange color measured from the base material was taken as a reference. When the figure is examined, and the colors of the weld zone at the 60% infill ratio and the reference sample are compared, it is understood that the color changes are regular. The values are close to each other (between 78.9–82.2 values). This is because the black color is dominant and prevents the orange color from appearing in the zone. Similarly, the color change in the weld zone at the 20% infill ratio was measured as 70.5–80.8, and the color change at the 100% infill ratio was measured as 79.1–84.5. In the case of the 20% infill ratio, weld defects were high. Therefore, it was considered inappropriate to interpret the relationship between color measurement and weld strength. When the graph is examined, it is understood that the color change decreases as the strength increases in the joints where the weld zone was at 60% and 100% infill ratios. This shows that there is a relationship between weld strength and color change. Color changes occurring in samples printed at a 20% infill ratio in the weld zone were not considered when evaluating the weld strength.

In Figure 12, the black color measured from the base material was taken as a reference. When black color was taken as a reference for the weld zone at a 60% infill ratio, color changes (between 2.6–7.3 values) were obtained. Since the black color was dominant, it is relatively more straightforward to see the color change compared to the orange color. The relationship between color changes and the strength of the samples with a 20% infill ratio in the weld zone was not considered for the same reasons as explained in Figure 12. The differences between the color changes in the weld zone were determined at 20%, 60%, and 100% infill ratios, respectively, when ranked from the smallest to the largest (Figure 11 and Figure 12). It is thought that the reason for this is that the color measurement is affected by the voids in part depending on the infill ratio. In Figure 11 and Figure 12, the color changes of the welded parts with 60% and 100% infill ratios showed similar trends.

### 3.6. Evaluation of Hardness Results after FSW

**Figure 13:**
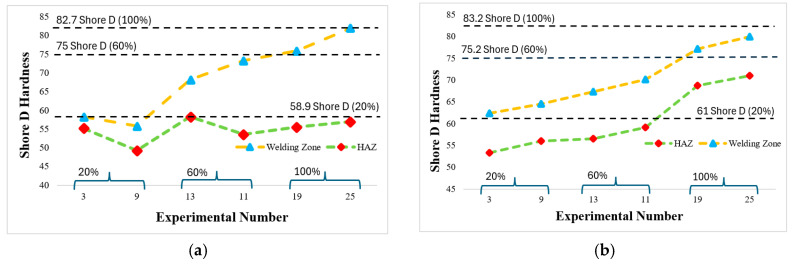
Shore D hardness evaluation (highest weld strength: 9, 11, and 25; lowest weld strength: 3, 13, and 19), (**a**) black filaman, (**b**) orange filaman.

Shore D hardness was measured to examine the effects of the heat generated during the FSW process on the weld and heat-affected zone (HAZ). The hardness values taken from the weld and HAZ were compared with the hardness values of the base material. The base material, source, and HAZ measurements with different infill ratios are given for the black filament in Figure 13a and for the orange filament in Figure 13b. As the infill ratio increased for both filaments, the hardness value increased. In addition, the hardness values obtained from the welding zone were higher than those from HAZ. In Figure 13a, for experiment number 11 (60%) and experiment number 25 (100%), the hardness values of the weld zone were very close to the base material. The orange filament (Figure 13b) showed hardness values taken from the weld zone that were lower than those taken from the base material. It can be said that this situation depends on the material flow during FSW and depolymerization after welding. It was determined that the weld strength increased as the hardness value increased in both colors.

### 3.7. Thermal Analysis during FSW

**Figure 14:**
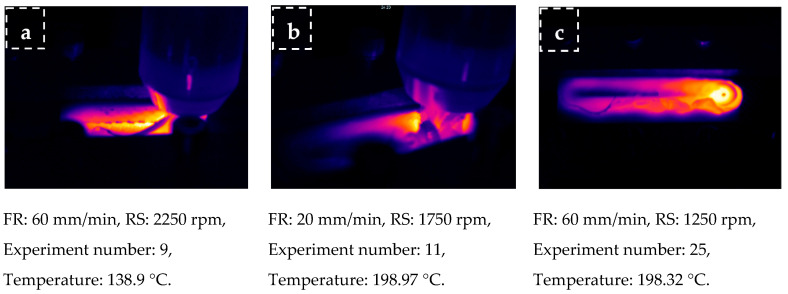
Thermal imaging of highest welding strength for welding zone 20% (**a**), welding zone 60% (**b**), and welding zone 100% (**c**).

**Figure 15:**
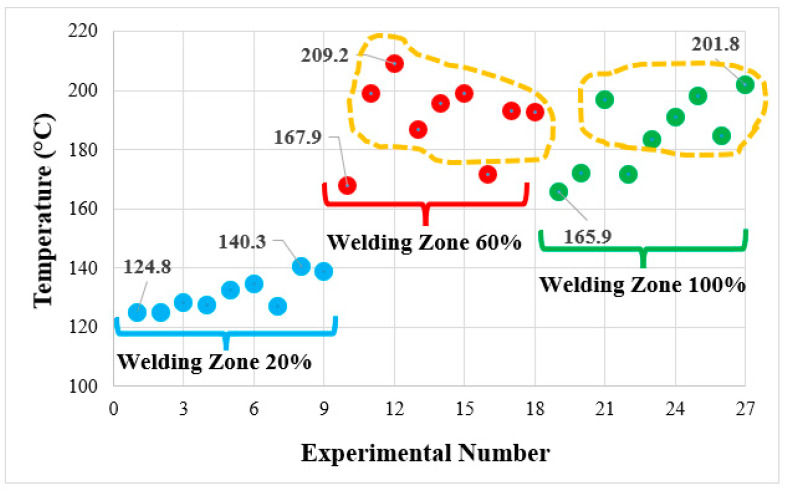
Maximum temperatures set for all experiments.

**Figure 16:**
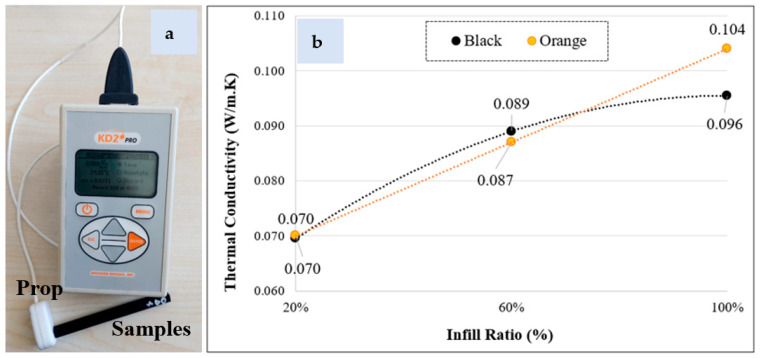
(**a**) Thermal conductivity measurement device. (**b**) Thermal conductivity depending on infill ratio and color.

Heat generation during FSW was examined using thermal images (Fluke Ti 32 thermal infrared camera). Figure 14 shows the heat images and maximum temperature values selected for the highest weld strength of parts with different infill ratios in the weld zone (a: 20%, b: 60%, and 100% infill ratios). According to the weld zone infill ratio, the temperatures determined for the lowest weld strengths were 124.8 °C, 167.9 °C, and 165.9 °C, respectively, and for the highest weld strengths were 138.9 °C, 198.9 °C, and 198.3 °C. The highest temperature in the weld zone was 209.2 °C for a 60% infill ratio and 201.8 °C for a 100% infill ratio. The highest temperatures measured during friction stir welding of parts with a 60% infill ratio, which is given in Figure 15, are relatively higher than those with a 100% infill ratio. When the average temperatures for 60% and 100% infill ratios were calculated, they were determined to be 190.5 °C and 185.0 °C, respectively. The reason for this is believed to be that the air voids in parts with a 60% infill ratio reduce heat conduction, thus increasing the temperature at the contact area. To determine the heat conduction phenomenon, samples were prepared with 20%, 60%, and 100% infill ratios using orange and black filaments, and their thermal conductivity coefficients were measured. For thermal conductivity measurement, the Decagon/KD2 Pro device equipped with a TR-1 sensor (2.4 mm diameter × 100 mm length) and operating according to the transient hot wire method, was utilized (Figure 16a). Measurements were conducted with five repetitions. The average thermal conductivity and temperature values are provided in Figure 14b. It was observed that the thermal conductivity values measured from samples varied between 0.007 and 0.104 W/mK for orange and between 0.007 and 0.096 W/mK for black, depending on the infill ratio and color.

The change in thermal conductivity values according to part infill ratio and color is given in Figure 16b. It has been observed that as the infill ratio increases, the thermal conductivity increases. This is because at a low infill ratio, the air in the spaces inside the part negatively affects the conductivity. In addition, while there was no change in thermal conductivity depending on color at the 20% infill ratio, thermal conductivity values changed as the infill ratio increased. Accordingly, it can be said that color pigments are effective in thermal conductivity and mechanical properties. Table 8 shows the highest, lowest, and optimum temperatures (highest weld strength) determined during FSW according to the infill ratio. Accordingly, it is seen that the values that give the highest weld quality are between the highest and lowest temperature values. This shows that the temperature required for the plasticization of the material depends on the process parameters, and the optimum temperature for material flow is between the highest and lowest temperatures.

## 4. Conclusions

The study examined the weldability of parts produced at low infill ratios with 3D printers using FSW and the effects of infill ratio changes in the weld zone on weld strength. The results obtained are given below.

Significant welding defects occurred due to material insufficiency in joining parts with a 20% infill ratio using FSW. The best weld strength was determined to be 17.83 MPa at a 20 mm/min feed rate, 1750 rpm rotational speed, and weld zone with a 60% infill ratio, and the welding efficiency was determined as 124.25% and 106.13% for black and orange, respectively. In parts with a 60% infill ratio, when the feed rate was 20 mm/min, the welding strength increased as the tool rotation speed increased.

It was observed that there was an improvement in joining parts with a 100% infill ratio in the weld zone using FSW compared to a 20% infill ratio. The best weld strength at a 100% infill ratio was obtained as 16.82 MPa at a feed rate of 60 mm/min and a rotation speed of 1250 rpm.

In general evaluation, welding efficiency was higher at a 60% infill ratio of the weld zone. It is thought that the reason for this is that the porous structure with a 60% infill ratio distributes the stresses better and delays the onset of fracture.

When hardness values were examined, they were determined from the highest to the lowest as base material, welding zone, and HAZ (heat-affected zone). It was determined that the reason for the increasing/decreasing tendency of the hardness change is similar to that of the weld strength change. When heat generation was examined, the lowest and highest temperatures at 60% and 100% infill ratios of the weld zone varied according to the FSW process parameters.

For both colors, black and orange, it was observed that the color changes in the samples with a 60% infill ratio in the weld zone were regular, and the values were close to each other. In this area where the welding performance was highest, it is understood from the color change that the material mixing in the welding area was stable.

**Suggestions**: PLA Plus, used as an experimental material in this study, is the same type of material, although its colors are different. For this reason, it is natural that weld strength curves show similar trends depending on color. The authors think that conducting a similar study with different materials in the future may make a difference in evaluating color-dependent weld strength.

To study the effect of filament colors on weld quality, color should be varied as a parameter while keeping the process parameters (tool rotation speed, feed rate, etc.) constant.

## Figures and Tables

**Table 1:** Physical and Mechanical Properties of PLA Plus [26].

Mechanical Properties	PLA Plus
Filament diameter (mm)	1.75
Color	Black/Orange
Tensile strength (MPa)	63
Density (g/cm^3^)	1.23
Brand	eSUN

**Table 2:** Experimental design for joining different infill ratios using FSW.

Base Material	Welding Zone	Pin Geometry	Feed Rate (mm/min)	Rotational Speed (rpm)
20%	20%	Triangle	20	1250
	60%		40	1750
	100%		60	2250

**Table 3:** Some studies on the welding of polymers by FSW.

Materials	Feed Rate (mm/min)	Rotational Speed (rpm)	Pin Geometry
PLA [8,31,43]	(20, 30, 40), (10, 14, 20), (3, 6, 9)	(700, 1400, 2000), (600, 1000, 1400)	Cylindrical, screw, and tapered
PLA Wood [14]	20, 40, 60	(1250, 1750, 2250)	Triangle, square, and screw
PLA/Al [30]	20, 30, 40	800, 1100, 1400	Cylindrical
HDPE [33,44,45]	(10, 25, 40), (45, 75, 115), (15)	(1250, 1600), (1500, 2000, 3000), (1200, 2000)	Cylindrical
PMMA [46]	25, 50	1250, 1600	Frustum
PE [47,48]	(12, 29, 44), (12.5, 25, 40)	(900, 1280, 1700), (1000)	Threaded (M10, M12, and M14)
PC [49]	2, 4, 6	600, 800, 1000	Cylindrical
ABS/PC [50]		800, 1200, 1600	Plane tapered, cylindrical threaded, and tapered threaded

**Table 4:** Pre-testing for the determination of initial parameters.

Experimental Number	Pin Geometry	Feed Rate (mm/min)	Rotational Speed (rpm)	UTS (MPa)
1	Square	20	2250	5.9 ± 0.19
2	Screw	20	2250	5.8 ± 0.26
3	Triangle	40	1250	7.6 ± 0.59

**Table 5:** UTS changes according to infill ratio and color.

Infill Ratio (%)	Color	UTS (MPa)	Elongation (%)	Hardness
20	Orange	16.80 ± 0.70	6.51 ± 0.36	61.0 ± 2.99
20	Black	14.35 ± 0.25	6.21 ± 0.32	58.9 ± 1.85
60	Orange	19.26 ± 0.25	4.73 ± 0.40	75.2 ± 1.35
60	Black	19.61 ± 0.93	7.35 ± 0.39	75.0 ± 0.60
100	Orange	42.32 ± 0.31	8.50 ± 0.61	83.2 ± 0.54
100	Black	53.07 ± 1.41	7.28 ± 0.88	82.7 ± 0.28

**Table 6:** Tensile results and weld efficiency of welded specimens with different weld zone infill ratios.

Number	Rotational Speed (rpm)	Feed Rate (mm/min)	Welding Zone Infill Ratio (%)	UTS (MPa)	Welding Efficiency (%)	
					Black	Orange
1	1250	20	20%	2.57 ± 0.69	17.96	15.34
2	1750	20	20%	2.74 ± 0.44	19.10	16.31
3	2250	20	20%	1.66 ± 0.17	11.63	9.93
4	1250	40	20%	2.50 ± 0.42	17.42	14.88
5	1750	40	20%	2.51 ± 0.13	17.53	14.98
6	2250	40	20%	1.87 ± 0.30	13.08	11.17
7	1250	60	20%	1.65 ± 0.20	11.54	9.86
8	1750	60	20%	2.60 ± 0.18	18.18	15.53
9	2250	60	20%	3.32 ± 0.35	23.17	19.79
10	1250	20	60%	16.11 ± 0.67	112.26	95.89
11	1750	20	60%	17.83 ± 0.68	124.25	106.13
12	2250	20	60%	17.76 ± 0.68	123.76	105.71
13	1250	40	60%	13.33 ± 0.60	92.89	79.35
14	1750	40	60%	15.28 ± 0.51	106.48	90.95
15	2250	40	60%	14.78 ± 0.23	103.00	87.98
16	1250	60	60%	14.23 ± 0.23	99.16	84.70
17	1750	60	60%	15.87 ± 1.02	110.59	94.46
18	2250	60	60%	16.82 ± 0.51	117.21	100.12
19	1250	20	100%	13.07 ± 0.89	77.14	65.89
20	1750	20	100%	13.58 ± 0.87	94.63	80.83
21	2250	20	100%	15.43 ± 0.11	107.53	91.85
22	1250	40	100%	15.13 ± 1.39	105.44	90.06
23	1750	40	100%	14.08 ± 0.51	98.12	83.81
24	2250	40	100%	16.71 ± 0.99	116.45	99.46
25	1250	60	100%	16.82 ± 0.90	117.21	100.12
26	1750	60	100%	14.15 ± 0.73	98.61	84.23
27	2250	60	100%	14.26 ± 0.32	99.37	84.88

**Table 7:** Measurement results of color parameters.

	Black			Orange			Welding Zone		
	20%	60%	100%	20%	60%	100%	20%	60%	100%
L	22.45	24.15	24.47	67.61	67.93	67.55	21.07	19.38	17.82
a	−0.56	−0.40	−0.43	43.75	43.19	43.23	2.834	−0.60	−0.62
b	−1.43	−1.16	−1.25	45.21	44.84	45.57	1.554	−1.50	−1.52

**Table 8:** The highest/lowest/optimum temperature determined during FSW.

Welding Zone Infill Ratio (%)	Temperature (°C)		
	Lowest	Highest	Best Welding Strength
20	124.8 (FR: 20 mm/min; RS: 1250 rpm)	140.3 (FR: 60 mm/min; RS: 1750 rpm)	138.9 (FR: 60 mm/min; RS: 2250 rpm)
60	167.9 (FR: 20 mm/min; RS: 1250 rpm)	209.2 (FR: 20 mm/min; RS: 2250 rpm)	198.97 (FR: 20 mm/min; RS: 1750 rpm)
100	165.9 (FR: 20 mm/min; RS: 1250 rpm)	2201.8 (FR: 60 mm/min; RS: 2250 rpm)	198.32 (FR: 60 mm/min; RS: 1250 rpm)

## Data Availability

The data that support the findings of this study are available from the corresponding author upon reasonable request.

## References

[1] Andronov V., Beránek L., Krůta V., Hlavůňková L., Jeníková Z. (2023). Overview and Comparison of PLA Filaments Commercially Available in Europe for FFF Technology. Polymers.

[2] Andalib H., Farahani M., Enami M. (2018). Study on the new friction stir spot weld joint reinforcement technique on 5754 aluminum alloy. Proc. Inst. Mech. Eng. Part C J. Mech. Eng. Sci..

[3] Tabasi M., Farahani M., Givi M.B., Farzami M., Moharami A. (2016). Dissimilar friction stir welding of 7075 aluminum alloy to AZ31 magnesium alloy using SiC nanoparticles. Int. J. Adv. Manuf. Technol..

[4] Sato Y., Nelson T., Sterling C., Steel R., Pettersson C.-O. (2005). Microstructure and mechanical properties of friction stir welded SAF 2507 super duplex stainless steel. Mater. Sci. Eng. A.

[5] Ramirez A.J., Juhas M.C. (2003). Microstructural evolution in Ti-6Al-4V friction stir welds. Materials Science Forum.

[6] Barlas Z., Uzun H. (2010). Microstructure and mechanical properties of friction stir butt welded dissimilar pure copper/brass alloy plates. Int. J. Mater. Res..

[7] Hajideh M.R., Farahani M., Ramezani N.M. (2018). Reinforced dissimilar friction stir weld of polypropylene to acrylonitrile butadiene styrene with copper nanopowder. J. Manuf. Process..

[8] Vidakis N., Petousis M., Mountakis N., Kechagias J.D. (2022). Material extrusion 3D printing and friction stir welding: An insight into the weldability of polylactic acid plates based on a full factorial design. Int. J. Adv. Manuf. Technol..

[9] Bagheri A., Azdast T., Doniavi A. (2013). An experimental study on mechanical properties of friction stir welded ABS sheets. Mater. Des..

[10] Tiwary V.K., Padmakumar A., Malik V.R. (2022). Investigations on FSW of nylon micro-particle enhanced 3D printed parts applied to a Clark-Y UAV wing. Weld. Int..

[11] Inaniwa S., Kurabe Y., Miyashita Y., Hori H. Application of friction stir welding for several plastic materials. Proceedings of the 1st International Joint Symposium on Joining and Welding.

[12] Derazkola H.A., Simchi A., Lambiase F. (2019). Friction stir welding of polycarbonate lap joints: Relationship between processing parameters and mechanical properties. Polym. Test..

[13] Moochani A., Omidvar H., Ghaffarian S.R., Goushegir S.M. (2019). Friction stir welding of thermoplastics with a new heat-assisted tool design: Mechanical properties and microstructure. Weld. World.

[14] Koçar O., Anaç N., Palaniappan S.K., Doğan M., Siengchin S. (2024). Effect of process parameters on the mechanical behavior of additively manufactured and FSW joined PLA wood sheets. Polym. Compos..

[15] Nergizhan A., Koçar O., Altuok C. (2024). Investigation of The Weldability of PLA Plus Sheets with Different Infill Ratios by Friction Stir Welding. Gazi Univ. J. Sci. Part C Des. Technol..

[16] Valerga A.P., Batista M., Salguero J., Girot F. (2018). Influence of PLA filament conditions on characteristics of FDM parts. Materials.

[17] Vosynek P., Navrat T., Krejbychova A., Palousek D. (2018). Influence of process parameters of printing on mechanical properties of plastic parts produced by FDM 3D printing technology. MATEC Web Conf..

[18] Frunzaverde D., Cojocaru V., Bacescu N., Ciubotariu C.-R., Miclosina C.-O., Turiac R.R., Marginean G. (2023). The influence of the layer height and the filament color on the dimensional accuracy and the tensile strength of FDM-printed PLA specimens. Polymers.

[19] Wittbrodt B., Pearce J.M. (2015). The effects of PLA color on material properties of 3-D printed components. Addit. Manuf..

[20] Pandžić A., Hodžić D., Milovanović A. Influence of material colour on mechanical properties of PLA material in FDM technology. Proceedings of the 30th DAAAM International Symposium.

[21] Gao G., Xu F., Xu J., Liu Z. (2022). Study of Material Color Influences on Mechanical Characteristics of Fused Deposition Modeling Parts. Materials.

[22] Brown J. How Does Color Affect the Welding Process?. https://laserplasticwelding.com/how-does-color-affect-the-welding-process.

[23] Incorporated. P.S 3 Types of Plastic Used in 3D Printing. https://www.polymersolutions.com/blog/plastic-in-3d-printing/.

[24] Kyutoku H., Maeda N., Sakamoto H., Nishimura H., Yamada K. (2019). Effect of surface treatment of cellulose fiber (CF) on durability of PLA/CF bio-composites. Carbohydr. Polym..

[25] Karakuş S. (2023). Design and Manufacturing of a Two-Stage Reduction Gearbox with 3D Printers. Int. J. 3D Print. Technol. Digit. Ind..

[26] eSUN PLA+. https://www.esun3d.com/pla-pro-product/.

[27] Torrado A.R., Roberson D.A. (2016). Failure analysis and anisotropy evaluation of 3D-printed tensile test specimens of different geometries and print raster patterns. J. Fail. Anal. Prev..

[28] Huang Y., Meng X., Xie Y., Wan L., Lv Z., Cao J., Feng J. (2018). Friction stir welding/processing of polymers and polymer matrix composites. Compos. Part A Appl. Sci. Manuf..

[29] Buffa G., Baffari D., Campanella D., Fratini L. (2016). An innovative friction stir welding based technique to produce dissimilar light alloys to thermoplastic matrix composite joints. Procedia Manuf..

[30] Kumar R., Singh R., Ahuja I. (2019). Mechanical, thermal and micrographic investigations of friction stir welded: 3D printed melt flow compatible dissimilar thermoplastics. J. Manuf. Process..

[31] Sharma A.K.R., Roy Choudhury M., Debnath K. (2020). Experimental investigation of friction stir welding of PLA. Weld. World.

[32] Wang F., Li W., Shen J., Hu S., Dos Santos J. (2015). Effect of tool rotational speed on the microstructure and mechanical properties of bobbin tool friction stir welding of Al–Li alloy. Mater. Des..

[33] Mostafapour A., Azarsa E. (2012). A study on the role of processing parameters in joining polyethylene sheets via heat assisted friction stir welding: Investigating microstructure, tensile and flexural properties. Int. Phys. Sci..

[34] Mendes N., Neto P., Simão M., Loureiro A., Pires J. (2016). A novel friction stir welding robotic platform: Welding polymeric materials. Int. J. Adv. Manuf. Technol..

[35] Azarsa E., Mostafapour A. (2014). Experimental investigation on flexural behavior of friction stir welded high density polyethylene sheets. J. Manuf. Process..

[36] Mostafapour A., Taghizad Asad F. (2016). Investigations on joining of Nylon 6 plates via novel method of heat assisted friction stir welding to find the optimum process parameters. Sci. Technol. Weld. Join..

[37] El Rayes M.M., Soliman M.S., Abbas A.T., Pimenov D.Y., Erdakov I.N., Abdel-mawla M.M. (2019). Effect of feed rate in FSW on the mechanical and microstructural properties of AA5754 joints. Adv. Mater. Sci. Eng..

[38] Senthil S., Kumar M.B. (2022). Effect of tool rotational speed and traverse speed on friction stir welding of 3D-printed polylactic acid material. Appl. Sci. Eng. Prog..

[39] Wu H., Chen Y.-C., Strong D., Prangnell P. (2015). Stationary shoulder FSW for joining high strength aluminum alloys. J. Mater. Process. Technol..

[40] Kumar P.S., Chander M.S. (2021). Effect of tool pin geometry on FSW dissimilar aluminum alloys-(AA5083 & AA6061). Mater. Today Proc..

[41] Meyghani B., Awang M. (2022). The influence of the tool tilt angle on the heat generation and the material behavior in friction stir welding (FSW). Metals.

[42] Hattingh D., Van Niekerk T., Blignault C., Kruger G., James M. (2004). Analysis of the FSW force footprint and its relationship with process parameters to optimise weld performance and tool design. Weld. World.

[43] Kumar R., Singh R., Ahuja I.S. (2020). Joining of 3D printed dissimilar thermoplastics with consumable tool through friction stir spot welding: A case study. Encyclopedia of Renewable and Sustainable Materials.

[44] Bozkurt Y. (2012). The optimization of friction stir welding process parameters to achieve maximum tensile strength in polyethylene sheets. Mater. Des..

[45] Saeedy S., Besharati Givi M. (2010). Experimental application of friction stir welding (FSW) on thermo plastic medium density polyethylene blanks. Eng. Syst. Des. Anal..

[46] Aghajani Derazkola H., Simchi A. (2018). Experimental and thermomechanical analysis of friction stir welding of poly (methyl methacrylate) sheets. Sci. Technol. Weld. Join..

[47] Rezgui M.-A., Ayadi M., Cherouat A., Hamrouni K., Zghal A., Bejaoui S. (2010). Application of Taguchi approach to optimize friction stir welding parameters of polyethylene. EPJ Web Conf..

[48] Arici A., Selale S. (2007). Effects of tool tilt angle on tensile strength and fracture locations of friction stir welding of polyethylene. Sci. Technol. Weld. Join..

[49] Vidakis N., Petousis M., David C., Sagris D., Mountakis N., Moutsopoulou A. (2024). The impact of process parameters and pin-to-shoulder ratio in FSW of polycarbonate: Welding forces and critical quality indicators. Int. J. Adv. Manuf. Technol..

[50] Kumar S., Roy B.S. (2024). Effect of different tool pin geometries on force, torque, microstructure and mechanical properties of friction stir welded acrylonitrile butadiene styrene and polycarbonate joints. Int. J. Interact. Des. Manuf..

[51] Anaç N. (2023). The mechanical properties of dissimilar/similar polymer materials joined by friction stir welding. Heliyon.

[52] Miklečić J., Zeljko M., Lučić Blagojević S., Jirouš-Rajković V. (2024). The Effect of Polyacrylate Emulsion Coating with Unmodified and Modified Nano-TiO2 on Weathering Resistance of Untreated and Heat-Treated Wood. Polymers.

[53] Nguyen V.D., Hao J., Wang W. (2018). Ultraviolet weathering performance of high-density polyethylene/wood-flour composites with a basalt-fiber-included shell. Polymers.

[54] Gao X., Zhang D., Qi S., Wen X., Su Y. (2019). Mechanical properties of 3D parts fabricated by fused deposition modeling: Effect of various fillers in polylactide. J. Appl. Polym. Sci..

[55] Yuan J., Tian J., Chen C., Chen G. (2020). Experimental investigation of color reproduction quality of color 3D printing based on colored layer features. Molecules.

[56] Bellehumeur C.T., Bisaria M., Vlachopoulos J. (1996). An experimental study and model assessment of polymer sintering. Polym. Eng. Sci..

[57] Aydemir C., Yenidoğan S., Karademir A., Arman E. (2017). Effects of color mixing components on offset ink and printing process. Mater. Manuf. Process..

[58] Kadhum A., Al-Zubaidi S., Abdulkareem S.S. (2023). Effect of the Infill Patterns on the Mechanical and Surface Characteristics of 3D Printing of PLA, PLA+ and PETG Materials. ChemEngineering.

[59] Kim Y., Fujii H., Tsumura T., Komazaki T., Nakata K. (2006). Three defect types in friction stir welding of aluminum die casting alloy. Mater. Sci. Eng. A.

[60] Mehta K., Astarita A., Carlone P., Della Gatta R., Vyas H., Vilaça P., Tucci F. (2021). Investigation of exit-hole repairing on dissimilar aluminum-copper friction stir welded joints. J. Mater. Res. Technol..

[61] Devaiah D., Kishore K., Laxminarayana P. (2018). Optimal FSW process parameters for dissimilar aluminium alloys (AA5083 and AA6061) Using Taguchi Technique. Mater. Today Proc..

[62] Shinde R.D., Rathi M.G. (2016). Optimization of FSW process parameter to achieve maximum tensile strength of aluminum alloy AA6061. Int. Res. J. Eng. Technol..

[63] Palanivel R., Koshy Mathews P., Murugan N. (2013). Optimization of process parameters to maximize ultimate tensile strength of friction stir welded dissimilar aluminum alloys using response surface methodology. J. Cent. S. Univ..

